# Interest linkage models between new farmers and small farmers: Entrepreneurial organization form perspective

**DOI:** 10.1371/journal.pone.0292242

**Published:** 2023-10-03

**Authors:** Qiang Liu, Junjie Ma, Liancui Wu

**Affiliations:** 1 Research Academy for Rural Revitalization of Zhejiang Province, Zhejiang A&F University, Hangzhou, China; 2 Institute of Ecological Civilization, Zhejiang A&F University, Hangzhou, China; 3 College of Economics and Management, Zhejiang A&F University, Hangzhou, China; Zhejiang Gongshang University, CHINA

## Abstract

Improving the interest linkage models between new farmers and small farmers is an important measure to realize the organic connection between small farmers and modern agricultural development. Based on the survey data of 572 new farmers in 16 provinces in China, this study uses the ordered probit model to empirically analyze the impact of entrepreneurial organization form on the interest linkage models between new farmers and small farmers. The results show that: (1) The choice of different entrepreneurial organization forms such as individual operation, cooperative operation, partner operation and company operation by new farmers will significantly affect the degree of interest linkage and then the linkage models. Partner operation and company operation have significantly improved the tightness of interest linkage between new farmers and small farmers. (2) The form of entrepreneurial organization significantly impacts the interest linkage between new farmers and small farmers. The higher the stability of entrepreneurial organization form, the closer the interest linkage and the more significant the impact on the interest linkage models. This effect remains significant after considering potential endogeneity issues and robustness tests. (3) In addition, further research also found significant regional differences and group differences in the impact of entrepreneurial organization form on the new farmers and small farmers’ interest linkage models. The impact of the western region is more significant than that of the eastern and central regions, and government entrepreneurship support policies can significantly strengthen the interest linkage models. The research results of this paper have vital reference significance for exploring the path of agricultural modernization under the "big country with small farmers".

## 1. Introduction

Agriculture is the foundation of the country. The report of the 19th National Congress of the Communist Party of China and the government’s Central No. 1 Document have repeatedly focused on the development of new farmers, such as family farms, farmer cooperatives, agricultural leading enterprises, and the group of individuals who have returned to rural areas for entrepreneurial activities, encouraging new farmers and small farmers to build a “community of interests.” After years of policy implementation, China has explored a variety of interest linkage models between new farmers and small farmers, including new order models, shareholding cooperation models, service-driven models, and multi-level integration models, *etc*. [1–3]. Nevertheless, the current model exhibits several issues, including issues related to its reliability, such as loose connections and a lack of stability in contractual relationships. Therefore, it is necessary to guide the formation of a closer relationship of interests between new farmers and small farmers, and promote the effective connection between small farmers and the development of modern agriculture [4].

According to the difference in the closeness of interest linkage between new farmers and small farmers, the interest connection model can be divided into loose type, semi-close type and tight type. However, at present, due to the lack of an effective connection mechanism, the interests between new farmers and small farmers in China are mainly loosely and semi-closely connected. There are still many problems in the connection of interests between them, mainly in the lack of an interest adjustment mechanism, and the connection method is relatively loose; the construction of interest protection mechanism lags, and the stability of the contract relationship is not strong; the interest distribution mechanism is unreasonable, and existence of government over-regulation [5–7]. Aiming at the problems existing in the interest linkage between new farmers and small farmers, studies have discussed them from two dimensions of interest competition and relationship coordination. It is suggested that the interests of both parties should be taken into account, the awareness of social responsibility of new farmers should be enhanced, and the voice of small farmers should be improved so as to support the strong and strengthen the weak and small [8, 9].

As research progresses, the organizational form of new farmers’ entrepreneurship has evolved into a prominent area of investigation. The study found that the form of entrepreneurial organization has a scale effect that measures the closeness of interest linkages, and the rationality of the choice of entrepreneurial organization form is directly related to the economic benefits obtained by new farmers [10, 11]. Different entrepreneurial organization forms have unique advantages in different agricultural fields and production processes. Appropriate entrepreneurial organization form can promote the effective of interest connection between new farmers and small farmers [12–14]. However, the existing studies have not further explored the impact of entrepreneurial organization form on the interest linkage models of new farmers and small farmers, nor have they compared the impact of different entrepreneurial organization forms, which will affect the formulation and implementation of policies.

Under the national conditions of "big country with small farmers", there is a compelling need to comprehensively examine the influence of entrepreneurial organization form on the connection mode of interests between new farmers and small farmers. The carry out of study is conducive to promoting the organic connection between small farmers and modern agricultural development, promoting the establishment of a long-term cooperation mechanism for agricultural management entities, and improving China’s agricultural modernization system. In addition, it is also conducive to gradually breaking the dual structure of rich and poor, narrowing the income gap, continuously improving the income level of low-income small farmers, and more actively promoting common prosperity. At present, the interest linkage tends to be stable, but the linkage mechanism needs to be improved. Analyzing from the perspective of new farmers’ entrepreneurial organization forms has far-reaching significance for building a more effective interest linkage mechanism, realizing the organic connection between small farmers and modern agriculture, and promoting the high-quality development of agriculture and rural areas [15–19].

Therefore, based on the sample survey data of 572 new farmers in 16 provinces in China, this study theoretically and empirically analyzes the impact of entrepreneurial organization form on the interest linkage models between new farmers and small farmers, and explores the regional differences and group differences of the influence. The development of the research can provide a theoretical explanation for the innovation of the new farmers’ entrepreneurial organization form, and provide a reference for the choice of interest connection models. It is of great practical significance to improve the closeness of the interest connection between new farmers and small farmers, stabilize the production cooperation relationship and promote the construction of new farmers and small farmers’ interest connection model system.

## 2. Theoretical analysis framework

### 2.1 Interest linkage model comparison between new farmers and small farmers

New farmers are operators and managers engaged in modern agricultural production. Guiding new farmers and small farmers to establish a stable and effective interest linkage mechanism is crucial to promoting small farmers’ income [20, 21]. The key to building a stable and effective interest linkage model is to reduce transaction costs. Generally speaking, the higher the transaction costs, the more stable the interest linkage model is needed to maintain the cooperative relationship between new farmers and small farmers [22]. Studies have found that information asymmetry, bounded rationality, asset specificity, opportunism, *etc*. will all affect the transaction costs of interest linkage of new farmers and small farmers, thereby affecting the stability and effectiveness of the interest linkage model [23–25]. In addition, the entrepreneurial organization form of new farmers’ is also an important factor that affects the pattern of interest linkage between them.

According to the three dimensions of new farmers’ entrepreneurial years and entrepreneurial scale, employment relationship and cooperative relationship, and local government entrepreneurship support policies, the interest linkage models of new farmers and small farmers can be divided into three different models, that is, loose type, semi-close type and tight type. Table 1 shows the differences, advantages and disadvantages of different models.

**Table 1:** Comparison of different interest linkage models between new farmers and small farmers.

Models		Loose type	Semi-close type	Tight type
Duration and scale of entrepreneurship		Short entrepreneurial period, with a relatively small scale	Relatively long entrepreneurial period, with a certain scale	Long entrepreneurial period, with a large scale
Employment and cooperation relationship		No stable employment relationship, few or short time cooperation	Short-term employment or signing purchase and sale contract	Formal employment or shareholding cooperation, with a long period
Local government entrepreneurship support policies		Insufficient support and less policy dividends	Relatively strong support, with certain subsidies	Strong support, with more subsidies
Evaluation	Advantages	Independent negotiation, freedom and flexibility, low transaction costs	Relatively clear rights and obligations	Stable cooperative relationship, with win-win situation
	Disadvantages	No fixed cooperation, greatly influenced by policies and markets	Unstable partnership, easy to cause a "prisoner’s dilemma"	Opportunism, high transaction costs

Source: Compiled by the author

### 2.2 The choice of new farmer’s entrepreneurial organization form and interest linkage models

**Fig 1:**
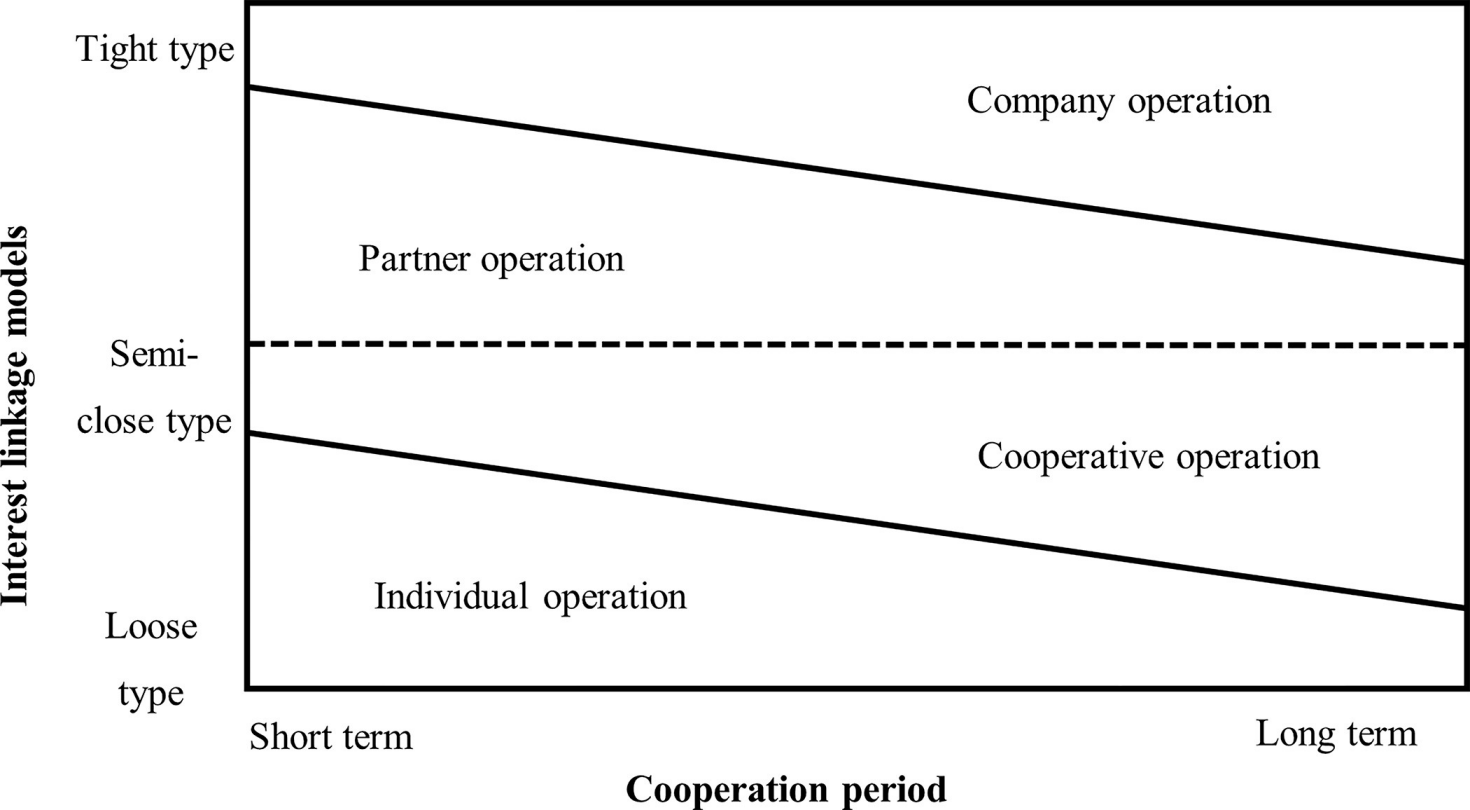
The relationship between entrepreneurial organization form and the interest linkage of new farmers and small farmers. Source: Compiled by the author.

Entrepreneurial organization form is a framework formed by division of labor and collaboration, which reflects the production and cooperation relationship among organization members [26]. According to different ways of entrepreneurship, the entrepreneurial organization forms of new farmers can be divided into four types, including individual operation, cooperative operation, partner operation and company operation. Different entrepreneurial organization forms have different impacts on the stability of the interest linkage between new farmers and small farmers, which will affect the choice of different interest linkage models for new farmers [27]. Fig 1 shows the relationship between the new farmers’ entrepreneurial organization form and the interest linkage of new farmers and small farmers.

Individual operation form. Individual operation is a relatively simple form of entrepreneurial organization. New farmers engage in agricultural production and self-management on an individual or family basis, the production cooperation period between new farmers and small farmers is relatively short, and the interests between them are not closely connected, which is suitable for small-scale agricultural production. In this form, the cooperation between new farmers and small farmers is mostly realized through limited markets, lacking a stable and effective cooperation foundation, and the interest is relatively loosely linked.

Cooperative operation form. Cooperative operation is a form of entrepreneurial organization for new farmers to provide production management services and information consulting services as the main content. Under the operation of cooperatives, new farmers and small farmers sign service contracts to clarify their respective rights and obligations, and build a group size based on transaction frequency. It is easy to form a long-term and stable cooperative relationship, and the interest is relatively closely linked [28, 29].

Partner operation form. Partner operation is an organizational model in which new farmers and small farmers work together to share benefits. Under partnership management, new farmers and small farmers are co-owners of the organization, and the cooperation between them is relatively stable, usually with long-term and stable cooperation agreements, and the interest is closely linked [30].

Company operation form. Company management is a cooperation model with a high degree of marketization. Under the management of the company, new farmers and small farmers have a stable employment relationship or long-term cooperative relationship. The two have relatively clear agreements on the division of rights and responsibilities, risks and benefits, and the interest is more closely linked [31, 32].

## 3. Data source and variable description

### 3.1 Data sources

The empirical research data of this study come from the sample survey of new farmers in 16 provinces in China in 2021, including Zhejiang, Anhui, Gansu, Guangdong, Guangxi, Hainan, Hebei, Henan, Hubei, Hunan, Jiangsu, Jiangxi, Shanghai, Tianjin, Chongqing and Sichuan. All the surveyed new farmers are adult household heads. The survey adopted random stratified sampling method. In each province, two agricultural counties were selected, and within each county, two townships with a primary focus on agriculture were chosen. Approximately 10 representative new farmers were selected from each township. To ensure the representativeness of the sample, the selection of new farmers was primarily based on random sampling from lists registered with the County Agricultural Department or the Industrial and Commercial Bureau, including family farms, cooperatives, and agricultural entrepreneurs, *etc*. A total of 32 counties, 64 towns, and 585 new farmers were surveyed. After excluding samples with missing key information, 572 valid samples were obtained. The survey was conducted in the form of questionnaires. During the survey, the staff of the local township agricultural station first contacted the new farmers, and then the researchers conducted a questionnaire survey. The main contents of the survey include basic information of new farmers (e.g. personal information, family information, *etc*.), entrepreneurial status (e.g. years of entrepreneurship, entrepreneurial scale, main business, *etc*.), status of cooperation between new farmers and small farmers (e.g. cooperation method, cooperation purpose, cooperation benefits, *etc*.), as well as relevant support policies of local governments for interest linkage of new farmers and small farmers. Besides, this study also collects data related to the regional socio-economic development through the Agricultural Bureau and Statistics Bureau where the new farmers are located.

According to the previous analysis, this study divides the interest linkage models of new farmers and small farmers into three types: loose type, semi-close type and tight type. Table 2 shows the cross-statistical data of the new farmer’s entrepreneurial organization form and interest linkage models. From the perspective of the interest linkage models, the interest linkage between new farmers and small farmers is still at a relatively low level, where the loose type accounts for 43.71%, and the semi-close type accounts for 35.31%. From the perspective of differences in entrepreneurial organization forms, individual operation and cooperative operation mainly adopt a loose interest-linked model, accounting for 59.48% and 57.41%, respectively. However, partner operation and company operation mainly adopt the semi-close interest linkage model, accounting for 43.27% and 49.59%, respectively. This is consistent with the theoretical analysis in Section 2.2.

**Table 2:** The entrepreneurial organization form and interest linkage model of new farmers.

Entrepreneurial Organization Form	Loose type	Semi-close type	Tight type	Total
Individual operation	69	32	15	116
Cooperative operation	93	36	33	162
Partner operation	48	74	49	171
Company operation	40	61	22	123
Total	250	203	119	572

Source: According to survey data

### 3.2 Variable description and statistics

Explained variable. According to the classification of the interest linkage models of new farmers and small farmers, combined with the actual research situation, in this study, loose type, semi-close type and tight type are assigned as 1, 2 and 3, respectively. The new farmers have significant differences in the choice of different interest linkage models, and the proportion of choosing the loose type is relatively high.

Explanatory variables. That is, four types of entrepreneurial organization forms. In this study, individual operation, cooperative operation, partner operation and company operation are assigned a value of 1, and other corresponding forms are assigned a value of 0. Different entrepreneurial organization forms reflect different production and organizational relationships, and the proportion of new farmers choosing different entrepreneurial organization forms is relatively close.

Control variables. Referring to the existing studies [1, 21, 33], entrepreneurial supports, entrepreneurial experiences, entrepreneurial purposes, characteristics of entrepreneurs, and production characteristics are 5 main factors that affect the entrepreneurial behavior among new farmers and are likely to influence the interest linkage models between new farmers and small farmers. According to the survey data, in this study, variables such as the effect of entrepreneurial support policies, the difficulty in obtaining entrepreneurial support, the entrepreneurial period, *etc*. are controlled from these 5 dimensions respectively. Additionally, provincial dummy variables are also controlled in the empirical analysis.

Variable descriptions and descriptive statistics are shown in Table 3.

**Table 3:** Variable descriptions and statistics.

Variable type	Variable name	Variable description and assignment	Mean	Standard deviation
**Explained variable**	Interest linkage models	1 = Loose type, 2 = Semi-close type, 3 = Tight type	1.771	0.771
**Explanatory variables**	Individual operation	1 = Individual operation, 0 = Others	0.203	0.402
	Cooperative operation	1 = Cooperative operation, 0 = Others	0.283	0.450
	Partner operation	1 = Partner operation, 0 = Others	0.299	0.458
	Company operation	1 = Company operation, 0 = Others	0.215	0.411
**Control variables**	**Entrepreneurial supports**			
	Effect of entrepreneurial support policies	1 = No effect; 2 = little effect; 3 = general effect; 4 = great effect; 5 = very great effect	3.108	1.139
	Difficulty in obtaining entrepreneurial support	1 = Very difficult; 2 = Difficult; 3 = Fair; 4 = Easy; 5 = Very easy	2.126	0.986
	**Entrepreneurial experiences**			
	Entrepreneurial period	Year	9.559	7.949
	**Entrepreneurial purposes**			
	Collaborate to reduce production cost	1 = Yes; 0 = No	0.378	0.485
	Collaborate to improve product quality	1 = Yes; 0 = No	0.570	0.496
	**Entrepreneurial characteristics**			
	Number of family farming labor force	No.	2.035	1.155
	Number of labor force with college degree or above	No.	1.528	0.962
	**Production characteristics**			
	Buying agricultural insurance	1 = Yes; 0 = No	0.729	0.468
	Expanding the scale of operation	1 = Yes; 0 = No	0.715	0.474
	Mainly planting	1 = Yes; 0 = No	0.790	0.560
	Mainly agricultural products processing	1 = Yes; 0 = No	0.385	0.487

Source: According to survey data

## 4. Model estimation and interpretation

### 4.1 Empirical model construction

In order to quantitatively analyze the impact of entrepreneurial organization form on the new farmers and small farmers’ interest linkage models, this study chooses the ordered probit model for empirical analysis. In the equation, the explained variable *Y*_*i*_ is the interest linkage models. The general form of the model is as follows:

$$Y_{i}^{*}=\beta X_{i}+\varepsilon_{i},\;E[\varepsilon_{i}|X_{i}]=0,\;\varepsilon_{i}\in (0,\;\delta_{i}^{2})$$ (1)

$$Y_{i}=\{\begin{array}{l} 1\;\mathrm{if}\;Y_{i}^{*}\in (-\infty ,\mu_{1})\\ 2\;\mathrm{if}\;Y_{i}^{*}\in [\mu_{1},\mu_{2})\\ 3\;\mathrm{if}\;Y_{i}^{*}\in [\mu_{2},+\infty ] \end{array}$$ (2)

In the equation, *Y*_*i*_ is an ordered value within the range of {1, 2,…k}, which represents the interest linkage models between new farmers and small farmers, 1 = loose type, 2 = semi-close type, 3 = tight type; $Y_{i}^{*}$ is a latent variable of interest linkage models of the new farmers and small farmers. *X*_*i*_ represents the influencing factor vector of the interest linkage models, which mainly includes four key explanatory variables of individual operation, cooperative operation, partner operation and company operation, as well as other factors related to entrepreneurial supports, entrepreneurial experiences, entrepreneurial purposes, entrepreneurial characteristics and production characteristics. The estimated equation is:

$$Prob(Y_{i}=1)=P(Y_{i}^{*}<\mu_{1})=\Phi (\mu_{1}-\beta X_{i})$$ (3)

$$Prob(Y_{i}=2)=P(\mu_{1}<Y_{i}^{*}<\mu_{2})=\Phi (\mu_{2}-\beta X_{i})-\Phi (\mu_{1}-\beta X_{i})$$ (4)

$$Prob(Y_{i}=3)=P(\mu_{2}\leq Y_{i}^{*})=1-\Phi (\mu_{2}-\beta X_{i})$$ (5)

In the equation, Φ(·) is the cumulative density function of the standard normal distribution. β is the parameter vector to be estimated. *ε*_*i*_ is the random error subject to the standard normal distribution, and *μ*_*i*_ (j = 1, 2) is the threshold.

### 4.2 Benchmark regression results

The empirical estimation results are shown in Table 4. In Table 4, Model 1, Model 2, Model 3 and Model 4 are the estimation results of different entrepreneurial organization forms. From the estimated results, the four entrepreneurial organization forms of individual operation, cooperative operation, partner operation and company operation all have a significant impact on the interest linkage models. The influence coefficients are -0.399, -0.205, 0.414 and 0.446, respectively. It can be seen that with the improvement of the stability of entrepreneurial organization form, the influence coefficient turns from negative to positive, and the interests of new farmers and small farmers are becoming more and more closely linked.

**Table 4:** Estimation results of benchmark model.

Variables	Model 1	Model 2	Model 3	Model 4
**Entrepreneurial Organization Form**				
Individual operation	-0.398*** (0.131)			
Cooperative operation		-0.206* (0.114)		
Partner operation			0.414*** (0.106)	
Company operation				0.446*** (0.106)
**Entrepreneurial supports**				
Effect of entrepreneurial support policies	0.068 (0.045)	0.068 (0.045)	0.063 (0.045)	0.075* (0.045)
Difficulty in obtaining entrepreneurial support	-0.050 (0.051)	-0.048 (0.051)	-0.040 (0.051)	-0.055 (0.052)
**Entrepreneurial experiences**				
Entrepreneurial period	0.003 (0.006)	-0.000 (0.006)	-0.000 (0.006)	-0.001 (0.006)
**Entrepreneurial purposes**				
Collaborate to reduce production cost	0.168* (0.102)	0.210** (0.102)	0.199** (0.102)	0.199* (0.102)
Collaborate to improve product quality	0.376*** (0.102)	0.356 (0.102)	0.357*** (0.102)	0.348*** (0.103)
**Entrepreneurial characteristics**				
Number of family farming labor force	-0.081* (0.044)	-0.064 (0.044)	-0.074* (0.044)	-0.067 (0.044)
Number of labor force with college degree or above	0.084 (0.051)	0.094* (0.051)	0.107** (0.051)	0.083 (0.051)
**Production characteristics**				
Buying agricultural insurance	0.183* (0.108)	0.135 (0.108)	0.124 (0.108)	0.156 (0.108)
Expanding the scale of operation	0.041 (0.112)	0.049 (0.112)	0.036 (0.112)	0.019 (0.113)
Mainly planting	0.163* (0.093)	0.155* (0.093)	0.120 (0.093)	0.164* (0.093)
Mainly agricultural products processing	0.168 (0.111)	0.186* (0.111)	0.185* (0.110)	0.092 (0.114)
**Provincial dummy variables**	Yes	Yes	Yes	Yes
LR Chi2	62.47***	56.36***	68.25***	70.72***
Pseudo *R*^2^	0.052	0.047	0.057	0.059

Notes: *, ** and *** denote significant at 10%, 5% and 1% levels, respectively. Standard errors in parentheses.

In particular, the regression coefficients for individual operation and cooperative operation on the interest linkage models between new farmers and small farmers demonstrate a negative effect, indicating that new farmers are more likely to opt for the loose interest linkage model under these two entrepreneurial organization forms. Under the loose interest linkage model, the cooperative relationship between new farmers and small farmers is relatively liberalized, which can minimize the cost of cooperation. In contrast, the impact coefficients of partner operation and company operation are positive, indicating that under these two entrepreneurial organization forms, new farmers are more inclined to choose the close interest linkage models. Under the circumstances, the cooperation relationship between the two is relatively stable, and the cooperation cost is also lower.

Within the scope of control variables, the variable entrepreneurial purpose significantly affects the interest linkage models between new farmers and small farmers. Both collaborate to reduce production cost and collaborate to improve product quality have a significant positive impact on the interset linkage models, indicating that the new farmers have a clear purpose in choosing the models of interest linkage. In addition, the government’s entrepreneurship support policy has a positive impact on the interest linkage models. However, the impact is not significant, and it is only significant in the company operation form. Among the characteristics of entrepreneurs, the number of college graduates and above has a significant positive impact, indicating that good education of new farmers can strengthen the interest linkage. Among the production characteristics, the new farmers who mainly focus on planting and agricultural product processing are more inclined to choose the tight interest linkage model.

### 4.3 Endogeneity test

Considering the endogeneity problem caused by the possible omitted variable bias, this study chooses the instrumental variable method for re-estimation. The selected instrumental variable is the convenience of transportation in the area where the new farmers are located, expressed in road mileage per square kilometer. The data comes from the National Bureau of Statistics. Generally speaking, the accessibility of regional transportation systems has a substantial impact on the choice of entrepreneurial organizational structure among new farmers. The more convenient the transportation, the better communication and cooperation among the organization members. At the same time, the convenience of regional transportation, as a geographical factor, does not directly affect the interest linkage models between new farmers and small farmers.

In this study, the IV-Probit estimation method was carried out, and the parameter estimation results are shown in Table 5. The weak instrumental variable identification test results show that the P values of AR under the four entrepreneurial organization forms are all significant at the 10% level, and the Wald chi-square value is significant at the 1% level. It can be considered that the instrumental variables selected in this study are reasonable. From the estimation results of the model, after considering possible endogenous problems, the impact of entrepreneurial organization form on the interest linkage between new farmers and small farmers is still robust.

**Table 5:** Estimation results of IV-Probit method.

Variables	Model 5	Model 6	Model 7	Model 8
Individual operation	-0.867*** (0.297)			
Cooperative operation		-0.909*** (0.249)		
Partner operation			0.714** (0.361)	
Company operation				1.126*** (0.162)
Control variables	Yes	Yes	Yes	Yes
Wald chi2	243.95***	240.34***	158.92***	839.72***

Notes: *, ** and *** denote significant at 10%, 5% and 1% levels, respectively. Standard errors in parentheses.

### 4.4 Robustness test

In this study, the type of new farmers is used as a proxy variable of entrepreneurial organization form, and the ologit model is used to re-estimate the impact of entrepreneurial organization form on the interest linkage models. Compared with the estimation results of IV-Probit model, the significance and direction of influence of the key variables remain stable, and the research results are highly reliable. See Table 6.

**Table 6:** Model robustness test results.

Variables	Model 5	Model 6	Model 7	Model 8
Individual operation	-0.623*** (0.220)			
Cooperative operation		-0.386** (0.194)		
Partner operation			0.696*** (0.180)	
Company operation				0.770*** (0.181)
Control variables	Yes	Yes	Yes	Yes
LR Chi2	64.83***	60.54***	71.62***	74.79***

Notes: *, ** and *** denote significant at 10%, 5% and 1% levels, respectively. Standard errors in parentheses.

### 4.5 Heterogeneity test

Considering differences in regional economic development levels and government entrepreneurship support policies, this study analyzes regional heterogeneity and government entrepreneurship support heterogeneity on the benchmark model.

Regional heterogeneity. In China, the problem of unbalanced regional economic development is prominent. Under different levels of economic development, there may be differences in the interest linkage degree of new farmers and small farmers. Therefore, this study divides the samples into three regions, the east, the middle and the west, and carries out sub-sample estimation respectively. The results are shown in Table 7. It can be seen from the estimated results that the impact of entrepreneurial organization form on the interest linkage models is more significant in the western region, partly significant in the central region, and not significant in the eastern region. One possible reason lies in that the economy in the eastern region is more diversified, and the difference in the degree of interest linkages is not significant, showing that the impact of entrepreneurial organization forms on the interest linkages is relatively small. While in the western region, the opposite is true. The degree of interest linkages vary significantly under different entrepreneurial organization forms, showing a relatively large impact. The survey also found that in the western region, due to a relatively lower degree of marketization and a larger number of small farmers, new farmers take into consideration the interests of small farmers to a greater extent when choosing their entrepreneurial organization form, while in the eastern region, due to the extensive mechanization, new farmers have less consideration for small farmers’ interests in their choice of entrepreneurial organization form.

**Table 7:** Differences in the impact of interest linkage models in different regions.

Variables	Eastern region	Middle region	Western region
Individual operation	-0.231 (0.284)	-0.417* (0.213)	-0.648*** (0.225)
Cooperative operation	-0.126 (0.197)	-0.190 (0.202)	-0.438** (0.213)
Partner operation	0.165 (0.168)	0.417* (0.230)	0.806*** (0.190)
Company operation	0.197 (0.179)	0.540*** (0.197)	0.853*** (0.195)

Notes: *, ** and *** denote significant at 10%, 5% and 1% levels, respectively. Standard errors in parentheses.

Heterogeneity of government entrepreneurship support policies. Discussing the differences in the impact of new farmers and small farmers under different entrepreneurship support policies will help to improve entrepreneurship support policies and cultivate new farmers groups. This study compares the difference in the impact of entrepreneurial organization form on the interest linkage models between new farmers and small farmers with and without government entrepreneurship support policies. The test results are shown in Table 8. It can be seen that compared with no entrepreneurship policy support, the government entrepreneurship support policy significantly strengthens this effect. In order to stabilize the interest linkages of new farmers and small farmers, it is necessary for the government to provide corresponding entrepreneurial support.

**Table 8:** Differences in the impact of interest linkage models under different entrepreneurship support policies.

Variables	Without government policy support	With government policy support
Individual operation	-0.523 (0.334)	-0.431*** (0.143)
Cooperative operation	0.013 (0.247)	-0.284** (0.131)
Partner operation	0.713** (0.303)	0.408*** (0.116)
Company operation	0.293 (0.252)	0.522*** (0.118)

Notes: *, ** and *** denote significant at 10%, 5% and 1% levels, respectively. Standard errors in parentheses.

## 5. Conclusion and discussion

Based on the sample survey data of 572 new farmers in 16 provinces in China, this study compares the differences between the new farmers and small farmers in the interest linkage models, and analyzes the impact of entrepreneurial organization form on the interest linkage models between new farmers and small farmers. The main conclusions are as follows: the organizational form of entrepreneurship has a significant impact on the interest linkage models between new farmers and small farmers. Compared with individual operation and cooperative operation, partner operation and company operation have a more significant positive impact on the interest linkage models. The impact in the western region is more significant than that in the eastern and central regions. In addition, the government’s entrepreneurship support policy has significantly strengthened the impact, and the new farmers with government policy support are more closely interest linked with small farmers.

In order to improve the mechanism for the interest linkage of new farmers and small farmers, and explore the path of agricultural modernization under the "big country with small farmers", the following suggestions are put forward:

Firstly, strengthening organizational construction is crucial to enhance the interest linkage between new farmers and small farmers. Due to the high transaction costs between new farmers and small farmers, the unstable contractual relationship, and unreasonable distribution of benefits, at present, the degree of organization of small farmers is still relatively low, and the interest linkage between new farmers and small farmers is still relatively loose in China. To strengthen the interest linkage between new farmers and small farmers, it is necessary to establish a cooperation platform and information sharing mechanism, provide effective information exchange, market connection and technical support, and promote exchanges and cooperation between new farmers and small farmers. Additionally, it is necessary to further improve the contractual relationship and benefit sharing mechanism, continuously reduce the cooperative costs, and improve the stability and effectiveness of cooperation between new farmers and small farmers.

Secondly, it is essential to improve the support policies for new farmers’ entrepreneurship, encouraging the establishment of long-term and stable cooperative relationships between new farmers and small-scale farmers. Specifically, it can be achieved by enhancing policy support for new farmers’ entrepreneurship at the levels of fiscal, finance, taxation and technological assistance. Encouraging the development of entrepreneurial organizational forms such as partner operation and company operation can help lower the technical barriers and financial costs associated with new farmers’ entrepreneurship, thus providing a better policy environment and external conditions for their entrepreneurial endeavors. Additionally, it is important to formulate and improve policy incentives according to the situation of new farmers driving small farmers. These incentives may include tax relief, preferential loans, and entrepreneurial subsidies, so as to effectively protect the rights and interests of small farmers, consolidate the "community of interests", and encourage the establishment of long-term and stable cooperation between new farmers and small farmers.

The research presented in this paper has important implications for developing countries with a large number of small farmers, especially for those transitioning from traditional agriculture to modern agriculture. Compared with the existing research, the existing research primarily focus on the impact of entrepreneurship on the welfare of new farmers [34–37], but pays less attention to the relationship between new farmers’ entrepreneurship and small farmers’ interest linkage in developing countries. Fortunately, there are still a few scholars who pay attention to the interest linkage between new farmers and small farmers. For example, researchers like Liverpool-Tasie, through a comparative analysis of 202 similar studies, argue that the interest linkage between small and medium enterprises and small farmers is stronger, and informal collaborations can effectively solve the problem of resource shortage for small farmers and make up for the shortcomings of the market [38]. This result is also supported by this study. Entrepreneurship among new farmers represents an upgrade and transformation of the farmer group, serving as an important pathway towards achieving agricultural modernization. However, in the face of a large number of small farmers, what kinds of entrepreneurial models the new farmers choose and how to strengthen their linkage with small farmers are still questions to be answered. The findings of this study suggest that entrepreneurial forms such as partner operation and company operation are more conducive to building a close interest connection with small farmers. This provides valuable insights for the new farmer cultivation policies in the future.

In addition, it should be noted that the interest linkage between new farmers and small farmers is a dynamic and evolving process, with varying applicability across different time periods, countries, and regions. Due to the impact of the COVID-19 pandemic, this study relied on cross-sectional data for analysis. In the future, it is necessary to further deepen the relevant research and strengthen the evaluation of policies related to the interest linkage between new farmers and small farmers.

## Supporting information

S1 Data (ZIP)
